# Combination of Exercise Training and SOD Mimetic Tempol Enhances Upregulation of Nitric Oxide Synthase in the Kidney of Spontaneously Hypertensive Rats

**DOI:** 10.1155/2020/2142740

**Published:** 2020-10-14

**Authors:** Pengyu Cao, Osamu Ito, Daisuke Ito, Rong Rong, Yang Zheng, Masahiro Kohzuki

**Affiliations:** ^1^Cardiovascular Disease Center, The First Hospital of Jilin University, Changchun, China; ^2^Department of Internal Medicine and Rehabilitation Science, Tohoku University Graduate School of Medicine, Sendai, Japan

## Abstract

Both exercise training (Ex) and superoxide dismutase (SOD) mimetic tempol have antihypertensive and renal protective effects in rodent models of several hypertensions. We recently reported that Ex increases nitric oxide (NO) production and the expression levels of endothelial and neuronal NO synthase (eNOS and nNOS) in the kidney and aorta of the spontaneously hypertensive rats (SHR) and normotensive Wistar–Kyoto rats (WKY). We also found that endogenous hydrogen peroxide (H_2_O_2_) upregulates the expression levels of eNOS and nNOS in SHR. To elucidate the mechanism of the Ex-upregulated NO system in the kidney, we examined the additive effect of Ex and tempol on the renal NO system in SHR and WKY. Our data showed that, in SHR, both Ex and tempol increase the levels of H_2_O_2_ and nitrate/nitrite (NOx) in plasma and urine. We also observed an increased renal NOS activity and upregulated expression levels of eNOS and nNOS with decreased NADPH oxidase activity. The effects of the combination of Ex and tempol on these variables were cumulate in SHR. On the other hand, we found that Ex increases these variables with increased renal NADPH oxidase activity, but tempol did not change these variables or affect the Ex-induced upregulation in the activity and expression of NOS in WKY. The SOD activity in the kidney and aorta was activated by tempol only in SHR, but not in WKY; whereas Ex increased SOD activity only in the aorta in both SHR and WKY. These results indicate that Ex-induced endogenous H_2_O_2_ produced in the blood vessel and other organs outside of the kidney may be carried to the kidney by blood flow and stimulates the NO system in the kidney.

## 1. Introduction

Exercise training (Ex) reduces the systemic blood pressure in human [1] and the hypertensive animal model including spontaneously hypertensive rats (SHR) [2, 3], Dahl salt-sensitive rats [4], and angiotensin II-infused rats [5]. In addition to antihypertensive effects, Ex offers renal protective effects through decreasing plasma creatinine and proteinuria and improving glomerular sclerosis, in rats with chronic renal failure [6, 7] or diabetic nephropathy [8]. However, the underlying mechanisms have not been fully understood.

Nitric oxide (NO) is a vasodilatory factor that can be synthesized by three isoforms of NO synthase (NOS): endothelial, neuronal, and inducible NOS (eNOS, nNOS, and iNOS), and it controls the systemic blood pressure and peripheral hemodynamics [9]. NO has various renal effects including the regulation of renal hemodynamics and the inhibition of renin secretion, tubular Na reabsorption, tubuloglomerular feedback, and sympathetic nerve activity [10, 11]. It has been reported that Ex increases the blood flow and NO production in the heart, aorta, and skeletal muscle [12, 13]. We recently reported that Ex increased NOS activity and expression levels of eNOS and nNOS in the aorta and kidney of SHR and normotensive Wistar–Kyoto rats (WKY) [14].

Superoxide anion (O_2_^−^) is generated by NADPH oxidase [15, 16] and metabolized into hydrogen peroxide (H_2_O_2_) by superoxide dismutase (SOD). Reactive oxygen species (ROS) and oxidative stress are elevated in SHR [17, 18] due to increased NADPH oxidase activity [19–21] and decreased SOD activity [22]. The SOD mimetic tempol lowers blood pressure and ameliorates the impaired vasodilatory responses in SHR [23, 24]. The expression levels of eNOS and nNOS and the NO production are elevated in the kidney and vessels of SHR compared to WKY [25–27]. In this regard, we recently reported that tempol further increases the mRNA levels of eNOS and nNOS in the kidney and aorta of SHR, and that H_2_O_2_ administered intravenously increases the levels of eNOS and nNOS in the kidney and aorta of WKY [27], suggesting that H_2_O_2_ could mediate the expression of NOS in the kidney and vessels of SHR. H_2_O_2_ may increase NOS expression and activity by improving the rate of gene transcription and mRNA processing and stability [28] and increasing the levels of phosphorylated eNOS at Ser1177 [27].

Previous studies reported that Ex increases the extracellular SOD (ecSOD) and eNOS expression in the mouse aorta [29], and the Ex-induced eNOS is mediated through H_2_O_2_ in the aorta of mice [30]. Considering that shear stress [31] and H_2_O_2_ [28] increases the expression of eNOS in cultured bovine aortic endothelial cells, we hypothesize that tempol enhances the effect of Ex on the NO system in the kidney of SHR. In the present study, we sought to clarify the mechanism of the Ex-upregulated NO system in the kidney through examining the additive effect of Ex and tempol on the renal NO system and oxidative stress in SHR and WKY.

## 2. Methods

### 2.1. Animal and Experimental Protocol

Five-week-old male SHR/Izm and WKY/Izm were obtained from SLC (Shizuoka, Japan). These rats were housed in the animal care facility at the Tohoku University School of Medicine under controlled temperature (24°C) and a 12 h light-dark cycle. All rats had free access to standard laboratory chow and water. All protocols involving rats were reviewed and received prior approval by the Animal Welfare Committee at the Tohoku University School of Medicine.

SHR and WKY were randomly divided into four groups (*n* = 10 in each group): control, Ex, tempol-treated (Tmp), and an Ex + Tmp. The treadmill running (20 m/min, 60 min/day, and 6 times/week) was performed with a rat treadmill (KN-73, Natsume Industries Co., Tokyo, Japan) to the Ex and Ex + Tmp groups, and tempol (1 mmol/l) was given to the Tmp and Ex + Tmp groups through drinking water for 8 weeks.

### 2.2. Blood Pressure Monitoring and Preparation of Plasma and Urinary Samples

The systolic blood pressure (SBP) was monitored by the tail-cuff method (Model UR-5000, Ueda, Tokyo, Japan). The rats were placed metabolic cages (Model ST; Sugiyama-General, Tokyo, Japan) individually on the day before the final experimental day, and urine samples were collected on ice over a period of 24 h. On the final experimental day, the rats were anesthetized with pentobarbital sodium (50 mg/kg, i.p.), and blood samples were collected by decapitation. These samples were centrifuged for 5 minutes at 1,500 rpm and separated from the sediments and stored at −80°C.

### 2.3. Plasma and Urinary Biochemical Variables Measurement

Creatinine and urea nitrogen were measured by a standard autoanalysis technique, (BML, Tokyo, Japan). H_2_O_2_ was measured using an Amplex Red Hydrogen Peroxide/Peroxidase Assay kit (Molecular Probes, OR, USA) [32]. Nitrate/nitrite (NOx) was measured spectrophotometrically by the Griess reagent method [33] using the Nitrate/Nitrite Colorimetric Assay Kit (Cayman Chemical Company, MI, USA).

### 2.4. Measurement of NADPH Oxidase, SOD, and NOS Activities

The preparation of tissue samples including the renal cortex (CO), outer medulla (OM), inner medulla (IM), and aorta was described previously [27]. The protein concentration of the samples was determined using the Bradford method [34]. The NADPH oxidase activity was assessed as an index of O_2_^−^ generation by the lucigenin-enhanced chemiluminescence method [14, 27] and presented as counts per minute (CPM)/mg of protein. The NADPH oxidase activity was examined in the CO, OM, and aorta, as it is undetectable in the IM. The SOD activity was measured by the Superoxide Dismutase Assay Kit (Cayman Chemical Company, MI, USA) utilizing a tetrazolium salt for detection of superoxide radicals generated by xanthine oxidase and hypoxathine. One unit (U) of SOD is defined as the amount of enzyme needed to exhibit 50% dismutation of the superoxidase radical. SOD activity was presented as U/g tissue protein. For the determination of NOS activity, the in vitro formation of NOx by each tissue was evaluated by using a colorimetric NOS activity assay kit (Oxford Biomedical Research, Inc., Rochester Hills, MI, USA) as described previously [14]. The NO production determined by NOx formation was linear with time and protein concentration. Data were presented as *μ*mol/g protein/unit time for the NOS activity.

### 2.5. Immunoblot

The level of NOS was examined by Western blot, as described previously [27, 35]. The relative intensities of the bands at the 140 kDa for eNOS, 155 kDa for nNOS, and 130 kDa for iNOS were quantified using the Image J software (version 1.40, National Institutes of Health, MD, USA) and normalized to *β*-actin.

### 2.6. Statistical Analysis

Data are presented as the means ± SEM. The comparison between the WKY control and SHR control used the one-way ANOVA. In WKY or SHR, compared with the control group, Ex, Tmp, and Tmp + Ex were all equivalent to the single factor intervention group, so one-way ANOVA was used. Subsequently, for the comparison between Ex, Tmp, and Tmp + Ex groups, data analysis was performed by Fisher's probability least significant difference (LSD) test for multiple comparisons after one-way ANOVA. In all analyses, a two-tailed *P* < 0.05 was considered as statistical significance. All statistical analysis data were performed using the SPSS 19 software (IBM Corp., Armonk, NY, USA).

## 3. Results

### 3.1. Effects of Ex and Tempol on Blood Pressure and Biochemical Variables in Plasma and Urine

The SBP, DBP, body weight, kidney weight, and plasma variables in the four groups of SHR and WKY are shown in Tables 1–3. The SBP, DBP, triglyceride, glucose, renal and aorta NADPH oxidase activities, and plasma and urinary H_2_O_2_ and NOx were significantly higher in the control SHR group than in the control WKY group (*P* < 0.01), but plasma creatinine and urea nitrogen and creatinine clearance were not significantly different between these control groups. In SHR, Ex and tempol significantly decreased the SBP, and there is an additive effect of the combination of Ex and tempol on the SBP. Ex significantly decreased the plasma creatinine and increased creatinine clearance (Ccr), which reflects the glomerular filtration rate (GFR), and tempol tends to increase Ccr (*P*=0.062). Other variables were not different among the four groups. In contrast to SHR, neither Ex nor tempol affected SBP or plasma variables in WKY.

**Figure 1:**
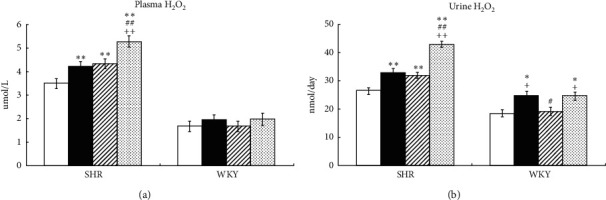
Effects of Ex and tempol on plasma and urinary H_2_O_2_ in SHR and WKY. The H_2_O_2_ in plasma (a) and urine (b) of SHR and WKY was compared among the control group (open bars), Ex group (closed bars), Tmp group (hatched bars), and Ex + Tmp group (checked bars) (*n* = 10 in each group). ^*∗*^*P* < 0.05 vs. the control group. ^*∗∗*^*P* < 0.01 vs. the control group. ^##^*P* < 0.01 vs. the Ex group. ^++^*P* < 0.01 vs. the Tmp group.

In SHR, Ex and tempol significantly increased the levels of H_2_O_2_ in plasma and urine (Ex: by 21% and 26%, *P* < 0.01; tempol: by 23% and 24%, *P* < 0.01) compared to controls. Notably, the combination of Ex and tempol further increased H_2_O_2_ in plasma and urine by 50% and 62% (*P* < 0.01) compared to the control group, and the differences between the Ex + Tmp and the Ex and Tmp only groups reached statistical significance (*P* < 0.01, respectively) (Figure 1). In WKY, Ex increased the level of H_2_O_2_ by 36% in urine (*P* < 0.05) compared to controls, but Tmp did not significantly change it. The combination of Ex and Tmp increased H_2_O_2_ by 35% in urine (*P* < 0.05) compared to controls, but the plasma and urinary H_2_O_2_ levels were not significantly different between the Ex + Tmp group and Ex only group (Figure 1).

**Figure 2:**
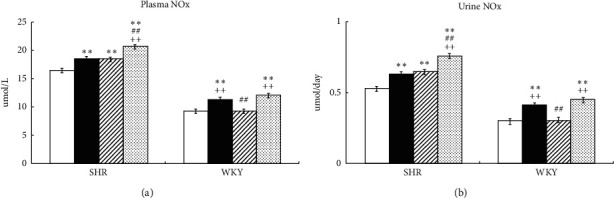
Effects of Ex and tempol on plasma and urinary NOx in SHR and WKY. The NOx in plasma (a) and urine (b) of SHR and WKY was compared among the control group (open bars), Ex group (closed bars), Tmp group (hatched bars), and Ex + Tmp group (checked bars) (*n* = 10 in each group). ^*∗*^*P* < 0.05 vs. the control group. ^*∗∗*^*P* < 0.01 vs. the control group. ^##^*P* < 0.01 vs. the Ex group. ^++^*P* < 0.01 vs. the Tmp group.

In SHR, Ex and Tmp significantly increased the NOx in plasma and urine, respectively (Ex: by 19% and 22%, *P* < 0.01; tempol: by 18% and 26% *P* < 0.01); the combination of Ex and Tmp further increased the NOx in plasma and urine to 38% and 47% compared to the controls (*P* < 0.01), and the NOx levels in the plasma and urinary were significantly higher in the Ex + Tmp group compared to the Ex and tempol groups (*P* < 0.01) (Figure 2). In WKY, Ex significantly increased the NOx in plasma and urine by 22% and 37%, but Tmp did not change them; the combination of Ex and Tmp increased the NOx in plasma and urine by 26% and 43% compared to controls (*P* < 0.01), but they were not significantly different in the Ex + Tmp group compared to the Ex group (Figure 2).

### 3.2. Effects of Ex and Tmp on NADPH Oxidase and SOD Activities

**Figure 3:**
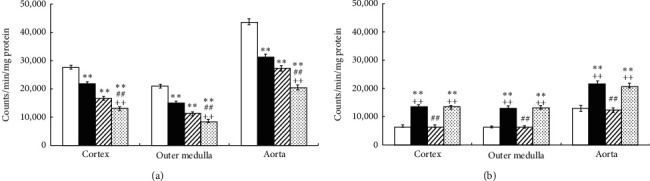
Effects of Ex and tempol on NADPH oxidase activity in SHR and WKY. The NADPH oxidase activity in the renal cortex, outer medulla, and aorta of SHR (a) and WKY (b) was compared among the control group (open bars), Ex group (closed bars), Tmp group (hatched bars), and Ex + Tmp group (checked bars) (*n* = 10 in each group). ^*∗*^*P* < 0.05 vs. the control group. ^*∗∗*^*P* < 0.01 vs. the control group. ^##^*P* < 0.01 vs. the Ex group. ^++^*P* < 0.01 vs. the Tmp group.

In SHR, Ex and Tmp significantly decreased the activity of NADPH oxidase in the CO, OM, and aorta. An additive effect of the combination of Ex and Tmp on the NADPH oxidase activity in these tissues was also found (Figure 3(a)). In WKY, Ex significantly increased the NADPH oxidase activity in the CO, OM, and aorta, but Tmp did not change the NADPH oxidase activity in these tissues; the combination of Ex and Tmp significantly increased the NADPH oxidase activity in the CO, OM, and aorta compared to controls, but not the Ex only group (Figure 3(b)).

**Figure 4:**
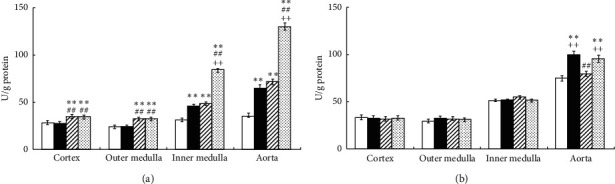
Effects of Ex and tempol on total SOD activity in SHR and WKY. The total SOD activity in the renal cortex, outer medulla, inner medulla, and aorta of SHR (a) and WKY (b) was compared among the control group (open bars), Ex group (closed bars), Tmp group (hatched bars), and Ex + Tmp group (checked bars) (*n* = 10 in each group). ^*∗*^*P* < 0.05 vs. the control group. ^*∗∗*^*P* < 0.01 vs. the control group. ^##^*P* < 0.01 vs. the Ex group. ^++^*P* < 0.01 vs. the Tmp group.

In SHR, Ex significantly increased SOD activity in the IM and aorta, but not in the CO and OM; Tmp significantly increased SOD activity in the kidney sections and aorta; there is an additive effect of the combination of Ex and Tmp on the SOD activity in the IM and aorta (Figure 4(a)). In WKY, Ex significantly increased SOD activity in the aorta, but not in the kidney sections; whereas Tmp did not change SOD activity in these tissues, and the combination of Ex and Tmp did not further increase SOD activity compared to the Ex only group (Figure 4(b)).

### 3.3. Effects of Ex and Tmp on the Level and Activity of NOS

**Figure 5:**
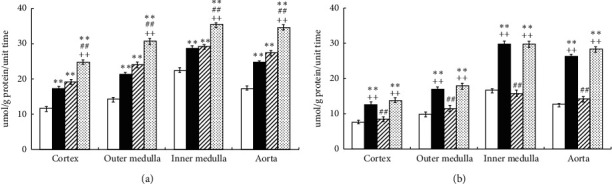
Effects of Ex and tempol on NOS activity in SHR and WKY. The NOS activity in the renal cortex, outer medulla, inner medulla, and aorta of SHR (a) and WKY (b) was compared among the control group (open bars), Ex group (closed bars), Tmp group (hatched bars), and Ex + Tmp group (checked bars) (*n* = 10 in each group). ^*∗*^*P* < 0.05 vs. the control group. ^*∗∗*^*P* < 0.01 vs. the control group. ^##^*P* < 0.01 vs. the Ex group. ^++^*P* < 0.01 vs. the Tmp group.

In SHR, Ex and Tmp significantly increased NOS activity in the kidney sections and aorta; an additive effect on NOS activity was observed for Ex and Tmp in the kidney sections and aorta (Figure 5(a)). In WKY, Ex significantly increased NOS activity in the kidney sections and aorta, but Tmp did not change the activity in these tissues; the combination of Ex and tempol increased NOS activity in the kidney sections and aorta compared to the controls, but not to the Ex group (Figure 5(b)).

**Figure 6:**
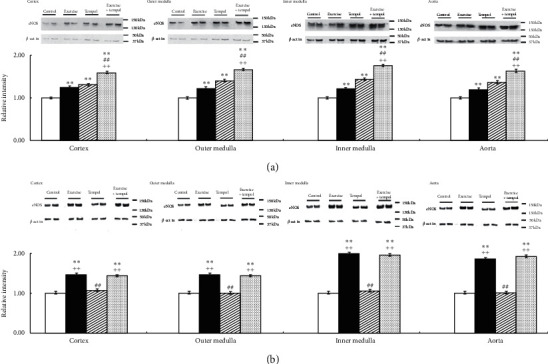
Effects of Ex and tempol on the protein levels of eNOS in SHR and WKY. The protein levels of eNOS in the renal cortex, outer medulla, inner medulla, and aorta of SHR (a) and WKY (b) were compared among the control group (open bars), Ex group (closed bars), Tmp group (hatched bars), and Ex + Tmp group (checked bars) (*n* = 10 in each group). Top panel shows representative immunoblots of eNOS, middle panel shows the immunoblots of *β*-actins, and bottom panel shows data of the densitometric analysis. ^*∗*^*P* < 0.05 vs. the control group. ^*∗∗*^*P* < 0.01 vs. the control group. ^##^*P* < 0.01 vs. the Ex group. ^++^*P* < 0.01 vs. the Tmp group.

The protein levels of eNOS and nNOS in the kidney sections and aorta were also examined. As shown in Figure 6, in SHR, Ex and Tmp significantly increased the level of eNOS in the kidney sections and aorta; and an additive effect of Ex and Tmp on the level of eNOS was found in the kidney sections and aorta (Figure 6(a)). In WKY, Ex significantly increased the protein level of eNOS in the kidney sections and aorta, but Tmp did not change it in these tissues. The combination of Ex and Tmp increased the eNOS protein level in the kidney sections and aorta compared to the controls, but not the Ex group (Figure 6(b)).

**Figure 7:**
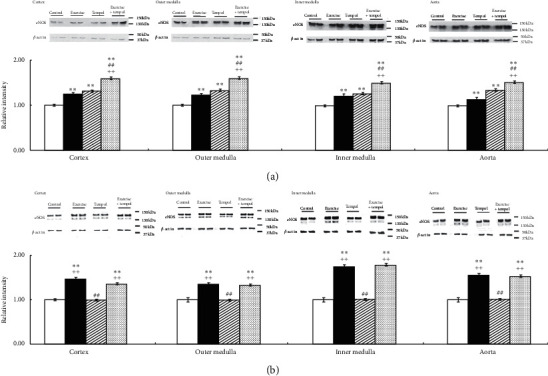
Effects of Ex and tempol on the levels of nNOS in SHR and WKY. The protein levels of nNOS in the renal cortex, outer medulla, inner medulla, and aorta of SHR (a) and WKY (b) were compared among the control group (open bars), Ex group (closed bars), Tmp group (hatched bars), and Ex + Tmp group (checked bars) (*n* = 10 in each group). Top panel shows representative immunoblots of nNOS, middle panel shows the immunoblots of *β*-actins, and bottom panel shows data of the densitometric analysis. ^*∗*^*P* < 0.05 vs. the control group. ^*∗∗*^*P* < 0.01 vs. the control group. ^##^*P* < 0.01 vs. the Ex group. ^++^*P* < 0.01 vs. the Tmp group.

As shown in Figure 7, in SHR, Ex and Tmp significantly increased the level of nNOS in the kidney sections and aorta; and there is an additive effect of Ex and Tmp on the protein level of nNOS in the kidney sections and aorta (Figure 7(a)). In WKY, Ex significantly increased the protein level of nNOS in the kidney sections and aorta, but Tmp did not in these tissues. The combination of Ex and Tmp increased the protein level of nNOS in the kidney sections and aorta compared to the controls, but not the Ex group (Figure 7(b)). Neither Ex or Tmp affected the iNOS level in the kidney sections and aorta of both strains of rats (data not shown).

## 4. Discussion

Although Ex increased the NOS activity and protein levels of eNOS and nNOS in the aorta and kidney of SHR and WKY, the mechanism of the Ex-upregulated NO system in the kidney has not yet been discovered. In the present study, we found that Ex increases concomitantly plasma and urinary H_2_O_2_ and NOx levels and renal NOS expression with decreased NADPH oxidase activity in SHR, but with increased NADPH oxidase activity in WKY. We also observed an additive effect of Tmp and Ex on these variables in SHR, but not in WKY.

In our previous study, we found that Tmp increases the plasma and urinary H_2_O_2_ and NOx levels in SHR, and endogenous H_2_O_2_ upregulates NO production [27]. The present study reported for the first time that both Ex and tempol increase the levels of plasma and urinary H_2_O_2_ and NOx in SHR, but not in WKY. Since the effects of Ex and tempol are similar, we think that Ex-induced NO production may also be related to the endogenous H_2_O_2_ in the plasma and urine of SHR.

We have shown that upregulation of O_2_^−^ in SHR may be dependent on elevated NADPH oxidase activity, and tempol increases the H_2_O_2_ level and decreases NADPH oxidase activity through the activation of SOD in SHR [27]. It has been shown that acute Ex also increases ROS including H_2_O_2_ [36], so we proposed that Ex may also increase the oxidative stress in SHR through a similar mechanism. However, we found that chronic Ex inhibits NADPH oxidase activity but increases SOD activity in SHR. Ito et al. also reported that Ex decreases an index of lipid peroxidation, thiobarbituric acid-reactive substances (TBARS) level in the urine of SHR [14]. These data suggest that Ex-induced upregulation of H_2_O_2_ and downregulation of NADPH oxidase activity might depend on increased SOD activity in SHR. Furthermore, we showed an additive effect of the combination of Ex and tempol on H_2_O_2_, NADPH oxidase, and SOD activities in the aorta of SHR. In addition, the previous study has reported that the catalase and glutathione peroxidase activities were not significantly increased in SHR [37]. Subsequently, the metabolism of plasma H_2_O_2_ could not be upregulated. Thus, in the present study, we also reported upregulation of H_2_O_2_ not only in plasma but also in urine of SHR.

It has been shown that Ex has no effect on the plasma and urinary TBARS levels in WKY [14]. In the present study, we demonstrated that Ex increases NADPH oxidase activity both in the kidney and aorta of WKY, but increases SOD activity only in the aorta. Furthermore, Ex-increased H_2_O_2_ is more significant in urine than in plasma of WKY. Taken together, Ex-increased plasma H_2_O_2_ may be carried to the kidney with blood flow and causes the significant increase in urine of WKY. In addition, the degree of Ex-induced NADPH oxidase activity in the kidney and aorta of WKY was still lower than that in the SHR control, and SOD activity of WKY control is two more times more than of the SHR control. We found that tempol had no effect on SOD activity not only in the WKY control group but also in the Ex-induced WKY group.

We previously reported that both Ex [14] and tempol [27] increase NOS activity and levels of eNOS and nNOS in the kidney and aorta of SHR. In recent studies, exogenous H_2_O_2_ increased the protein level of NOS in the kidney of WKY [27], and exogenous H_2_O_2_ increased eNOS phosphorylation in rats' aortas [36]. A key finding of the present study is the additive effects of Ex and tempol on NOS activity and NOS expression and plasma and urinary H_2_O_2_ levels in SHR. Furthermore, the Ex-increased NO system in the kidney and aorta of WKY is also accompanied with the increase in H_2_O_2_. Thus, endogenous H_2_O_2_ may be a mediator of Ex-induced NOS activity and NOS expression in the kidney and aorta [29, 31]. The lack of the additive effect of Ex and high dose (2 mmol/l) tempol on H_2_O_2_ and the NO system in SHR (data not shown) could be explained by the sufficiency of low dose of tempol on suppressing the oxidative stress in combination with Ex in SHR.

Fukai et al. reported that Ex-upregulated the expression levels of vascular eNOS and extracellular SOD in wild-type mice [31]. In the aorta, the Ex-induced blood flow could enhance shear stress, leading to the increase of oxidative stress [12]; in response to the elevated oxidative stress, nuclear factor-erythroid-2-related factor 2 (Nrf2) is synthesized and translocated to the nucleus where it activates the transcription of several antioxidants including SOD [38]. On the other hand, Ex decreased the renal blood flow [39], and shear stress could not increase the SOD activity directly in the kidney. In agreement with these studies, we also found that Ex stimulates the SOD activity in aorta in both SHR and WKY, but not in CO and OM of SHR or the kidney sections of WKY. In the skeletal muscle, Ex also enhanced xanthine oxidase activity [40], resulting in the increase of O_2_^−^ and SOD activity. Taken together, Ex-increased endogenous H_2_O_2_ in the plasma and urine might be produced in the blood vessel and other organs including the skeletal muscle, but not the kidney. The endogenous H_2_O_2_ was produced by Ex-increased SOD activity and NADPH oxidase activity in the aorta and would be then carried to the kidney with blood flow and causes the increase of the renal NOS activity and expression in SHR and WKY. Further studies are needed to identify the nonrenal H_2_O_2_-mediated mechanism of Ex-induced NOS activity and expression in the kidney.

Ex increased NOS activity and expression in the kidney and aorta, which in turn improved numerous physiological effects of NO in SHR, including vasodilatory responses [41] and various renal effects including regulation of renal hemodynamics and inhibition of renin secretion, tubular Na^+^ reabsorption, tubuloglomerular feedback (TGF), and sympathetic nerve activity [10, 11, 42]. In this regard, we found that both Ex and tempol decrease SBP and increases Ccr, which reflects the glomerular filtration rate (GFR) in SHR, and the effects of the combination of Ex and Tmp on these variables are additive. In consistent with the present results, the recent studies also reported that chronic running Ex upregulates renal NOS expression and normalizes renal NADPH oxidase, accompanied by the improvements of urinary albumin excretion [14], Ccr, glomerulosclerosis, podocyte injury, and tubulointerstitial injury in ZDF rats at an early stage of diabetic nephropathy [43]. It is also reported that tempol increases the GFR, Ccr [24], and the renal medullary blood flow by approximately 50% in SHR [19]. The renal nephron can be subdivided into at least 13 different segments; the thick ascending limb is not truly a single segment but is at least two segments with cortical and medullary thick ascending limbs that possess quite different characteristics. NO produced by NOS inhibits NaCl absorption by this segment [44]. Whether the effects of the NO bioavailability in the renal artery and subsequent branches are different need further research and demonstration.

It is widely accepted in recent years that not only NO but also H_2_O_2_ has the effect on vasodilation. H_2_O_2_ has been shown to be a major component of the endothelium-derived hyperpolarizing factor (EDHF) in several vascular beds in multiple species [45, 46], and EDHF is considered to be a major mechanism controlling blood pressure [47]. H_2_O_2_ could also cause interprotein disulfide bond in protein kinase G (PKG) I-*α*, which activates the kinase independent of the NO-cyclic guanosine monophosphate (cGMP) pathway and coupled to vasodilation [48]. Moreover, pharmacological inhibition of PKG attenuated H_2_O_2_-induced vasorelaxation in mice mesenteries [49]. Consist with the overall results, the antihypertensive and renal protective effects of Ex in SHR might partially depend on the improvement of NO bioactivity, which at least partially rely on the direct effect of H_2_O_2_ on vasorelaxation. However, other studies have yielded conflicting results that H_2_O_2_ revealed vasoconstrictor effects: endothelium-dependent contraction [50] or vasomotor tone [51, 52] effects depending on the different vascular beds and the experimental conditions.

In conclusion, the present study indicates that the combination of Ex and Tempol offers additive effects on the NOS activity and levels of eNOS and nNOS in the kidney with decreased renal NADPH oxidase activity and increased arterial SOD activity in SHR. We also demonstrate that the Ex increases the renal NO system with increased renal NADPH oxidase activity and arterial SOD activity in WKY. These results suggest that the Ex-upregulated NO system in the kidney is mediated through the increased endogenous H_2_O_2_ production in the blood vessel and other organs including the skeletal muscle but not the kidney.

## Figures and Tables

**Table 1:** The SBP and biochemical variables in SHR control and WKY control.

	WKY	SHR
	Control (*n* = 10)	Control (*n* = 10)
SBP (mmHg)	153 ± 8	225 ± 6^*∗∗*^
DBP (mmHg)	105 ± 4	130 ± 5^*∗∗*^
Body weight (g)	378 ± 16	386 ± 15
Kidney weight/body weight (g/kg)	6.21 ± 0.53	6.48 ± 0.27
Total cholesterol (mg/dl)	69 ± 4.55	64.40 ± 2.08
Triglyceride (mg/dl)	32.17 ± 3.27	53.00 ± 4.39^*∗∗*^
Free fatty acid (mEq/l)	0.24 ± 0.02	0.21 ± 0.01
Glucose (mg/dl)	177.17 ± 5.36	199.80 ± 5.62^*∗∗*^
Plasma H_2_O_2_ (umol/L)	1.71 ± 0.22	3.51 ± 0.18^*∗∗*^
Urine H_2_O_2_ (nmol/day)	19.73 ± 1.46	26.45 ± 1.24^*∗*^
Plasma NOx (umol/L)	9.78 ± 0.39	16.55 ± 0.39^*∗∗*^
Urine NOx (umol/day)	0.30 ± 0.03	0.53 ± 0.03^*∗∗*^
Renal cotex NADPH oxidase activity (counts/min/mg)	6624 ± 388	27743 ± 687^*∗∗*^
Renal outer medulla NADPH oxidase activity (counts/min/mg)	6362 ± 368	21038 ± 625^*∗∗*^
Aorta NADPH oxidase activity (counts/min/mg)	13352 ± 487	43644 ± 866^*∗∗*^
Plasma creatinine (mg/dl)	0.19 ± 0.01	0.17 ± 0.01
Creatinine clearance (ml/min)	2.15 ± 0.31	2.50 ± 0.51
Plasma urea nitrogen (mg/dl)	18.88 ± 0.63	17.86 ± 0.60

Values are means ± SEM. ^*∗*^*P* < 0.05 vs. the WKY control group. ^*∗∗*^*P* < 0.01 vs. the WKY control group.

**Table 2:** Effects of Ex and tempol on the SBP and biochemical variables in SHR.

	Control (*n* = 10)	Ex (*n* = 10)	Tmp (*n* = 10)	Ex + Tmp (*n* = 10)
SBP (mmHg)	225 ± 6	211 ± 4^*∗∗*^	212 ± 6^*∗∗*^	202 ± 4^*∗∗*#+^
DBP (mmHg)	130 ± 5	122 ± 5	125 ± 3	123 ± 3
Body weight (g)	386 ± 15	350 ± 23	366 ± 13	358 ± 19
Kidney weight/body weight (g/kg)	6.48 ± 0.27	5.93 ± 0.19	6.04 ± 0.29	6.12 ± 0.11
Total cholesterol (mg/dl)	64.40 ± 2.08	61.80 ± 2.35	59.20 ± 2.11	59.00 ± 2.25
Triglyceride (mg/dl)	53.00 ± 4.39	61.40 ± 4.66	51.80 ± 5.75	61.20 ± 5.04
Free fatty acid (mEq/l)	0.21 ± 0.01	0.20 ± 0.02	0.20 ± 0.02	0.18 ± 0.02
Glucose (mg/dl)	199.80 ± 5.62	187.80 ± 5.85	187.00 ± 7.50	193.40 ± 4.12
Plasma creatinine (mg/dl)	0.17 ± 0.01	0.11 ± 0.02^*∗∗*^	0.14 ± 0.02	0.08 ± 0.01^*∗∗*+^
Creatinine clearance (ml/min)	2.50 ± 0.51	4.57 ± 0.67^*∗∗*^	3.28 ± 0.46	5.82 ± 0.75^*∗∗*+^
Plasma urea nitrogen (mg/dl)	17.86 ± 0.60	17.34 ± 0.75	17.28 ± 0.73	16.78 ± 0.79

Values are means ± SEM. ^*∗*^*P* < 0.05 vs. the control group. ^*∗∗*^*P* < 0.01 vs. the control group. ^#^*P* < 0.05 vs. the Ex group. ^+^*P* < 0.05 vs. the Tmp group.

**Table 3:** Effects of Ex and tempol on the SBP and biochemical variables in WKY.

	Control (*n* = 10)	Ex (*n* = 10)	Tmp (*n* = 10)	Ex + Tmp (*n* = 10)
SBP (mmHg)	153 ± 8	155 ± 5	153 ± 6	154 ± 6
DBP (mmHg)	105 ± 4	108 ± 3	102 ± 4	103 ± 3
Body weight (g)	378 ± 16	359 ± 22	362 ± 12	356 ± 19
Kidney weight/body weight (g/kg)	6.21 ± 0.53	6.34 ± 0.33	6.03 ± 0.26	6.28 ± 0.17
Total cholesterol (mg/dl)	69 ± 4.55	69.80 ± 4.35	65.50 ± 4.39	64.60 ± 4.11
Triglyceride (mg/dl)	32.17 ± 3.27	37.80 ± 5.66	31.80 ± 5.75	34.20 ± 5.04
Free fatty acid (mEq/l)	0.24 ± 0.02	0.26 ± 0.02	0.26 ± 0.02	0.21 ± 0.02
Glucose (mg/dl)	177.17 ± 5.36	167.60 ± 7.03	179.80 ± 6.93	165.40 ± 7.12
Plasma creatinine (mg/dl)	0.19 ± 0.01	0.17 ± 0.01	0.18 ± 0.01	0.16 ± 0.01
Creatinine clearance (ml/min)	2.15 ± 0.31	2.45 ± 0.41	2.29 ± 0.35	2.64 ± 0.42
Plasma urea nitrogen (mg/dl)	18.88 ± 0.63	17.36 ± 0.69	20.75 ± 0.84	17.38 ± 0.78

## Data Availability

The data used to support the findings of this study are available from the corresponding author upon request.
